# Characterisation of the merozoite thrombospondin related anonymous protein (MTRAP) of *Plasmodium berghei* as a transmission-blocking antigen

**DOI:** 10.1590/0074-02760230217

**Published:** 2024-03-22

**Authors:** Xiomara Alexandra Gaitán, Juliana Calit, Irina Dobrescu, Marisé Solórzano Ramos, Alba Marina Gimenez, Daniel Youssef Bargieri

**Affiliations:** 1 Universidade de São Paulo, Instituto de Ciências Biomédicas, Departamento de Parasitologia, São Paulo, SP, Brasil

**Keywords:** Plasmodium, transmission, blocking, MTRAP

## Abstract

**BACKGROUND:**

Malaria is an infectious disease caused by protozoan parasites belonging to the genus *Plasmodium*. Human-to-human transmission depends on a mosquito vector; thus, the interruption of parasite transmission from humans to mosquitoes is an important approach in the fight against malaria. The parasite stages infectious to mosquitoes are the gametocytes, sexual stages that are ingested by the vector during a blood meal and transform into male and female gametes in the midgut. Immunity against sexual stage antigens expressed by gametocytes, gametes, and the zygote formed after fertilisation can interrupt the parasite sexual cycle in the mosquito. This transmission blocking immunity is mediated by specific antibodies ingested during the mosquito blood feed, inhibiting the parasite development in the midgut. Merozoite thrombospondin related anonymous protein (MTRAP) is a merozoite and gametocyte surface protein essential for gamete egress from erythrocytes and for parasite transmission to mosquitoes.

**OBJECTIVES:**

Here, we evaluated the potential of the *P. berghei* MTRAP to elicit antibodies with the ability to inhibit gamete fertilisation *in vitro*.

**METHODS:**

We expressed a soluble recombinant *Pb*MTRAP and used it to immunise BALB/c mice. The transmission blocking activity of the anti-r*Pb*MTRAP antibodies was tested through *in vivo* challenge experiments followed by *in vitro* conversion assays.

**FINDINGS:**

Immunisations with the r*Pb*MTRAP induced a strong antibody response and the antibodies recognised the native protein by Western Blot and IFA. Anti-r*Pb*MTRAP present in the blood stream of immunised mice partially inhibited gamete conversion into ookinetes.

**CONCLUSION:**

Our results indicate that antibodies to *Pb*MTRAP may reduce but are not sufficient to completely block transmission.

Malaria is a parasitic infectious disease that can be prevented, diagnosed, and treated. Nonetheless, as per the ‘Malaria Report 2022’ published by the World Health Organization (WHO),^1^ there were approximately 247 million cases of malaria in 2021, spanning across 85 countries with ongoing transmission. These numbers indicate an increment of 13 million cases between 2019 and 2021.

The transmission of malaria to humans occurs through the bites of infected *Anopheles* mosquitoes, and the symptomatic form of the disease arises from the rapid multiplication of asexual stages of *Plasmodium* species within red blood cells. Control efforts against malaria are partly focused on vector control. This involves employing insecticidal nets and administering long-lasting indoor residual insecticide sprays to shield humans from mosquito bites. However, reports of increased mosquito resistance to insecticides between 2010 and 2020 have raised concerns.^1^

While various approaches have been employed in the fight against malaria, vaccines have proven to be the most effective strategy in eradicating diseases. Currently, the availability of a licensed malaria vaccine is limited to Mosquirix^®^, which has been endorsed by the WHO for administration to infants in African countries affected by the deadliest species of malaria, *P. falciparum*. However, this vaccine’s effectiveness diminishes within four years, making it far from an ideal solution for malaria prevention.^2^

The complexity of *Plasmodium* life cycle and the scarce knowledge regarding antigenic targets of protective immunity have hindered vaccine development against malaria. Nonetheless, evidence suggests that the goal of creating an efficient vaccine against malaria is achievable. For instance, individuals residing in malaria-endemic regions, who face persistent exposure to malaria infection, develop a natural protective immunity that mitigates the severity of the disease, leading to a decrease in both morbidity and mortality rates.^3^ This phenomenon plays a crucial role in reducing disease transmission as certain antibodies target the surface antigens of gametes, zygotes and ookinetes, the first parasite stages formed in the mosquito midgut after a blood meal. Transmission reduction by natural immunity supports the development of transmission blocking vaccines (TBVs) targeting sexual stage antigens expressed on the surface of gametocytes/gametes (pre-fertilisation antigens), or zygotes/ookinetes (post-fertilisation antigens).^4^

The pre-fertilisation antigens that have received the most attention are P48/45 and P230, which are abundantly present on the gametocyte membrane. These antigens persist in the gametes and zygotes but are absent during the zygote’s transformation into ookinetes. P48/45 and P230 represent the most extensively studied gametocyte membrane proteins, as they become exposed upon the emergence of gametes from red blood cells (RBCs). On the other hand, P25 and P28 are located on the zygotes and ookinetes surfaces, being only expressed by parasites in the mosquito vector. Pfs25 is still being developed as a TBV candidate, while Pfs28 has been found as not effective by itself but enhances the transmission-blocking activity of antibodies against Pfs25.^4^

Another antigen that deserves attention in this context is the merozoite thrombospondin-related anonymous protein (MTRAP). MTRAP belongs to the TRAP family, which comprises transmembrane proteins characterised by the presence of one or more thrombospondin type-I repeat (TSR) domains. These proteins play a crucial role in parasite motility and invasion by interacting with the actin-myosin motor complex.^5^ ^,^ ^6^ MTRAP is localised at the micronemes and processed during merozoite invasion^7^ and its disruption is dispensable for *P. berghei* asexual blood stages but is essential for completion of the sexual cycle in the mosquito vector.^8^ *P. berghei* and *P. falciparum* MTRAP knock-out (KO) gametes fail to disrupt the parasitophorous vacuole membrane and remain trapped inside the parasitophorous vacuole, which results in a complete fertilisation block in mosquitoes.^8^ Due to its significant involvement in the *Plasmodium* sexual life cycle, MTRAP emerges as a potential target for the development of TBVs.

In this study, we assessed the ability of a recombinant *P. berghei* MTRAP protein to elicit specific antibodies in mice. Additionally, we investigated whether these antibodies could effectively inhibit the conversion of gametes into ookinetes through *in vivo* and *ex vivo* experiments.

## MATERIALS AND METHODS

*Protein expression and purification* - *Pb*MTRAP amino acids 26-562 sequence (PlasmoDB accession nº PBANKA_0512800), was codon optimised and cloned in pET28a for expression in *Escherichia coli* (GeneScript). Recombinant *E. coli* BL21 DE3 (Novagen) was cultivated at 37ºC in flasks containing Luria broth (LB) and kanamycin (30 µg/mL). The His-tagged recombinant *Pb*MTRAP (r*Pb*MTRAP) expression was induced at an OD_600_ of 0.6 with 0.1 mM isopropyl-β-D-thio-galactopyranoside (IPTG, Invitrogen) for 4 h. Bacteria were harvested, resuspended in lysis buffer (56 mM NaH_2_PO4, 128 mM NaCl, 1 mM PMSF and 0.2 mg/mL lysozyme, pH 7.0) and subjected to 12 cycles of sonication on ice with an ultrasonic processor (each cycle of 30 seconds and pulses with intervals of 40 seconds and intensity of 10%). Bacterial lysates were centrifuged and the supernatants were passed through a 0.22 µm filter and loaded to a Ni-NTA agarose resin (QUIAGEN). The resin was washed with 56 mM NaH_2_PO4, 128 mM NaCl and 10% glycerin and the bound protein was eluted with 0.5M imidazole. Purified protein was dialysed overnight to phosphate buffer saline (PBS, pH 7.4) at 4ºC in agitation. Protein sample was quantified by Bradford assay and analysed by sodium dodecyl sulphate polyacrylamide gel electrophoresis (SDS-PAGE).

*Mice immunisation and challenge* - BALB/c mice were bred and maintained at the Mouse Facility of the Parasitology Department, Institute of Biomedical Sciences, University of São Paulo, Brazil. All protocols using rodent model were approved by the Institutional Animal Care and Use Committee (CEUA) of the Institute (protocol number 85/2017) and the animals were handled according to the Brazilian College of Animal Experimentation guidelines.

The *P. berghei ANKA* and the *Ookluc* lines were stored as frozen stocks in liquid nitrogen or at -80ºC, and vial stocks were prepared as previously described.^9^ Mice were infected by intraperitoneal injection of 200-μL portions of thawed stocks, and the parasitaemia was evaluated through giemsa staining of thin blood smears counted by direct light microscopy with a 100× oil immersion objective (Nikon E200).

Four to six weeks old female BALB/c mice were subcutaneously (s.c.) immunised three times with the vaccine formulation of 10 µg r*Pb*MTRAP/adjuvant, at 3-week intervals. Control groups received only adjuvants or a saline solution.

For immunological assays, the recombinant protein was emulsified in equal volume of Incomplete Freund Adjuvant (IFA, SIGMA). One week after the last dose, the immunogenicity was evaluated by enzyme-linked immunosorbent assay (ELISA). Two weeks after the last dose, mice were euthanised and the sera was collected and stored at -20ºC for further analyses.

For immunisation/challenge schedules, mice were immunised with 10 µg r*Pb*MTRAP in combination with 50 µg Polyinosinic-polycytidylic acid [Poly (I:C)] (Invivogen). Immunogenicity was evaluated by ELISA three weeks after each dose. Three weeks after the last immunising dose, groups were challenged intravenously with 5×10^3^ erythrocytes parasitised with the *P. berghei Ookluc* line, obtained from previously infected donor mice. Parasitaemia and gametocytaemia were monitored daily after challenge infection by microscopic examination of stained blood smears. When the gametocytaemia reached 0.4%, the conversion assay was performed. Mice were euthanised when signs of severe disease/illness were observed.

*Immunogenicity test by ELISA* - Antibody titres against r*Pb*MTRAP were measured by ELISA. Briefly, high-binding plates (Corning, ref 3590) were incubated overnight with r*Pb*MTRAP (100 ng/well). Plates were washed three times with PBS-0.02% Tween 20 (PBS-T) and blocked for 2 h with PBS - 0.02% Tween - 1% BSA - 5% Skim-Milk. Mice sera were added at serial three-fold dilutions starting at 1:200, v:v, and incubated for 2 h in PBS-0.02% Tween - 0.25% BSA - 5% skimmed-milk. Plates were washed with PBS-T and secondary antibody HRP-conjugated anti-mouse IgG was diluted 1:2,000 and incubated for 2 h. Finally, after washing with PBS-T, 100 µL of revelation buffer (0.2 M Na_2_HPO_4_, 0.2 M citric acid, 0.10% of OPD and H_2_O_2_, pH 4,7) was added to each well for 15 min. The reaction was stopped with 50 µL of H_2_SO_4_ 4N, and the optical density (OD) was read at 490 nm using an ELISA plate reader (BioTek, ELx800). Antibody titres were determined as the log of the last serial dilution with OD > 0.1.

*Antibody purification* - IgG antibodies were purified from sera obtained from mice immunised with recombinant protein emulsified in IFA or adjuvant only. For IgG purification, a protein G-sepharose resin was washed with PBS and mixed 1:1, v:v, with the pool of serum of each mice group. The interaction was allowed for 2 h in agitation at 4ºC. Later, the matrix with the sample was placed on a glass column and washed with PBS. Antibodies were eluted with 0.1 M Glycine (pH: 3.0) and the reaction was neutralised with 1M Tris-HCL (pH: 8.0). Fractions were quantified using Nanodrop and dialysed against PBS in an Amicon filter (Millipore).

*Western blot analysis* - Red blood cells collected from mice infected with *Pb*ANKA WT were lysed with 0.15% saponin. Parasites were centrifuged, washed 3x with PBS, and RIPA buffer was used to obtain a parasite extract. 4 µg of total extract were subjected to SDS-PAGE electrophoresis under reduction conditions. The electro transference to nitrocellulose membrane was performed at 100 v for 2 h. After transference, the membrane was blocked (PBS - 0.05% Tween - 5% skimmed milk) for 2 h. Purified antibodies anti-Poly I:C e anti-*Pb*MTRAP were diluted in block solution 1:2,000 (1.5 mg/mL) and incubated overnight in agitation at 4ºC. After three washes, membrane was incubated with secondary antibodies Goat anti-Mouse-HRP (1:2,000), for 1 h in agitation. Revelation was performed with SuperSignal^®^ West Femto substrate (Thermo Scientific) and visualised in ChemiDoc.

*Immunofluorescence assay* - Gametocytes were obtained by Nycodenz gradient. Blood from five infected mice collected by cardiac puncture was diluted 1:4 in RPMI 1640 (pH: 7.2) at 37ºC. Gametocytes were isolated with Nycodenz 13.25% and centrifuged (500 g, 20 min at 37ºC without break). The interphase containing gametocytes was washed with RPMI 1640 (450 g, 10 min at 37ºC). Parasites in the pellet were fixed with 4% PFA - 0.0075% Glutaraldehyde for 1 h. After three washes with PBS and centrifugation at 2.400 g for 2 min, parasites were immobilised on a glass slide with cytospin (500 g). Fixed parasites were permeabilised (Triton X-100 0.1% diluted in PBS) for 10 min and then blocked with PBS-BSA 3% for 1 h at room temperature. After three washes, parasites were incubated with primary antibody (anti-Poly I:C and anti-*Pb*MTRAP) diluted 1:500, overnight at 4ºC. Anti-Mouse Alexa^®^ fluor 488 (Life Technology) was used as secondary antibody for 30 min at room temperature. Parasite nuclei were stained with DAPI and analysed by fluorescence microscopy.

*Conversion assay* - The ability of the antibodies to block ookinete formation was evaluated through conversion assays as described.^9^ *Ookluc* mutant parasites express the nanoluciferase gene under the control of the CTRP promotor, a gene expressed in the Ookinete stage, allowing quantification of conversion from gametocytes to ookinete through detection of the relative light units in a luminometer.^9^ Mice infected with *P. berghei Ookluc* transgenic parasites presenting gametocytaemia between 0.4 - 1% were used. Blood collected by cardiac puncture was maintained at 37ºC to prevent gametocyte activation, and then diluted 1:20 in ookinete medium containing the purified antibodies previously diluted in different concentrations. Each concentration was tested in triplicates per assay. The cultures were incubated at 21ºC for 24 h. For measurement of the ookinete formation, parasites were lysed and subsequently the substrate/lysis buffer for nanoluciferase (Nano-Glo luciferase assay system; Promega) was added and luminescence was detected in SprectraMax i3. The results are represented as the percentage of inhibition in the ookinete conversion, where the mean relative light unit (RLU) of the replicates was normalised to the mean of the replicates in the control group.

To perform conversion assays with non-purified antibodies, immunised mice were challenged with 5×10^3^ erythrocytes parasitised with the *Ookluc* line. When the gametocytaemia reached ~0.4% (approx. day 6 after challenge), a conversion assay was performed as described above. The results are represented as the percentage of inhibition in the ookinete conversion, where the mean RLU of the replicates are normalised to the gametocytaemias of each mouse and then divided by the mean of the replicates in the control group.

*Statistical analysis* - One-way analysis of variance (ANOVA) followed by Tukey’s honestly significantly different (HSD) test was used to calculate statistical significance. Differences were considered significant when *p*-values were ≤ 0.05. Prism 6 software (GraphPad Software Inc., LA Jolla, CA) was used for all tests and graphics.

## RESULTS

*Plasmodium berghei-MTRAP expression and purification* - A soluble recombinant form of *P. berghei* MTRAP (r*Pb*MTRAP) was successfully expressed in *E. coli*. To achieve this, the *E. coli* culture was induced with 0.1 mM IPTG and incubated at 37ºC for 4 h. The recombinant protein contained a histidine tag, facilitating subsequent purification and characterisation of r*Pb*MTRAP.

**Fig. 1:**
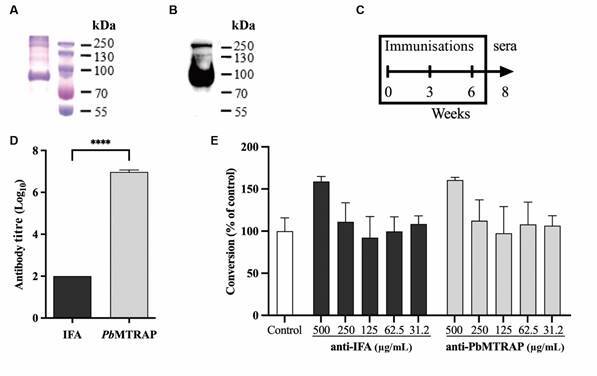
antibody response in mice immunised with recombinant *Plasmodium berghei* merozoite thrombospondin related anonymous protein (r*Pb*MTRAP) plus Incomplete Freund Adjuvant (IFA) adjuvant. (A) Sodium dodecyl sulphate-polyacrylamide gel electrophoresis (SDS-PAGE) under reducing conditions of 4 µg of the purified r*Pb*MTRAP stained with Coomassie blue. (B) Western blot using 2 µg of the recombinant protein. Antibodies used were monoclonal anti-histidine and anti-mouse IgG HRP-labeled, and detection was performed by electrochemiluminescence assay. (C) BALB/c mice were immunised s.c. with three doses 21 days apart with 10 µg of r*Pb*MTRAP in combination with IFA, and the sample and sera collection were performed according to the timeline described. (D) The IgG antibody titres in pooled sera against r*Pb*MTRAP analysed by enzyme-linked immunosorbent assay (ELISA) after the last immunising dose. Statistical analyses: unpaired t-test; ****p < 0.0001. (E) Conversion assay *in vitro* with purified antibodies. Blood samples from Ookluc-infected mice were diluted 1:20 in ookinete medium containing the purified antibodies previously diluted in the concentrations described. The results are expressed as the percentage of inhibition in the ookinete conversion ± standard deviation (SD) (n = 2, each with experimental triplicate).

The Ni-NTA purification process yielded a soluble protein with an electrophoretic migration pattern ranging from 70 to 100 kDa under reducing conditions with estimated > 90% purity (Fig. 1A). This observed pattern is consistent with the predicted molecular weight of approximately 70 kDa. To validate the successful purification of the recombinant protein, a western blot analysis was performed using anti-His antibodies that specifically recognise the hexa-histidine tag present in the recombinant protein. As anticipated, a prominent band at ~70 kDa was detected, further confirming the correct purification of the recombinant protein (Fig. 1B).

*rPbMTRAP immunogenicity and antibody specificity* - To evaluate the immunogenicity of the purified r*Pb*MTRAP, BALB/c mice were first immunised using IFA as adjuvant. The mice were administered three doses of the recombinant protein, and the levels of IgG anti-*Pb*MTRAP antibodies were measured by ELISA following the final immunisation (Fig. 1C). The antibody titres in the r*Pb*MTRAP group showed a statistically significant increase compared to the control group (p < 0.001), clearly indicating that immunisation with r*Pb*MTRAP effectively stimulated the production of specific antibodies in the immunised mice (Fig. 1D).

**Fig. 2:**
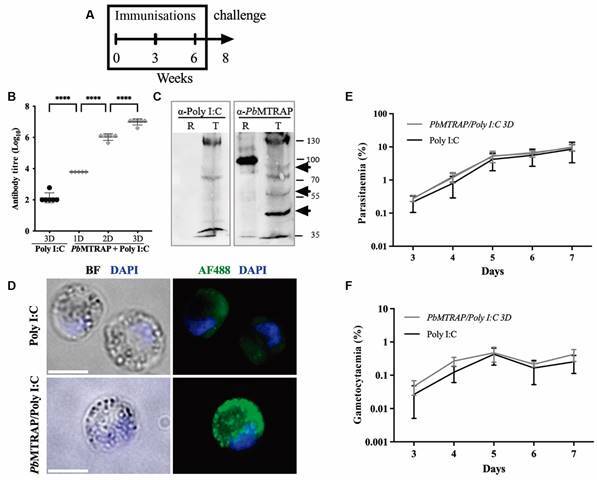
antibody response and functionality in mice immunised with recombinant *Plasmodium berghei* merozoite thrombospondin related anonymous protein (r*Pb*MTRAP) plus Poly (I:C). (A) Mice immunisation and challenge schedule: groups of five BALB/c mice were immunised s.c. with three doses 21 days apart with r*Pb*MTRAP (10 µg/dose) in the presence of the adjuvant Poly (I:C), or with Poly (I:C) alone. Two weeks after the last immunising dose, mice were infected with 5,000 asexual forms of Ookluc *P. berghei* parasites (Challenge). (B) Antibody response analysed by enzyme-linked immunosorbent assay (ELISA) three weeks after each immunising dose. Statistical analyses: One-way analysis of variance (ANOVA) followed by Tukey’s HSD test, ****p < 0.0001. (C) Western blot using 2 µg of the recombinant protein r*Pb*MTRAP (R) or *P. berghei* blood stage total extract (T). Antibodies used were polyclonal sera anti-*Pb*MTRAP, and anti-Poly (I:C) as control. (D) Immunofluorescence on fixed and permeabilised *P. berghei* ANKA gametocytes performed using pool of sera (diluted 1:500) from BALB/c mice immunised with r*Pb*MTRAP plus Poly (I:C) or adjuvant alone as a negative control. Secondary antibody was Alexa 488-labeled anti-mouse IgG (green) and nuclei were visualised by DAPI staining (blue). (E, F) Mice were challenged i.v. with 5×10^3^ red blood cells infected with *P. berghei* ANKA and parasitaemia and gametocytaemia were followed daily by blood smears.

In other experiments, the same immunisation protocol was performed, but changing the adjuvant to Poly (I:C) (Fig. 2A). To assess the antibody response, levels of IgG anti-*Pb*MTRAP antibodies were measured using ELISA after each immunisation. The results demonstrated that all immunised mice exhibited high specific antibody titres, which were detectable even after the first immunising dose. Furthermore, a dose-dependent response was observed, indicating a robust immune response to the r*Pb*MTRAP (Fig. 2B).

To determine the specificity of the anti-r*Pb*MTRAP antibodies generated in the immunised mice serum, we conducted a western blot to assess whether the antibodies could recognise the native *Pb*MTRAP protein in parasite extracts. We observed that the antibodies derived from the serum of immunised mice exhibited recognition of a specific band of approximately 85 kDa in the total extract of the parasite (Fig. 2C). Additionally, we detected two processed fragments with molecular weights of 55 kDa and 45 kDa, suggesting proteolytic cleavage events associated with the native *Pb*MTRAP protein as described for the *P. falciparum* MTRAP.^7^

To further investigate the ability of the anti-r*Pb*MTRAP antibodies to recognise the protein on the surface of the intact parasite, we conducted an immunofluorescence assay. Antibodies obtained from mice immunised with the r*Pb*MTRAP stained the surface of gametocytes, while antibodies obtained from control mice did not (Fig. 2D).

*Antibodies anti-rPbMTRAP reduce ookinete formation* - The ability of the generated antibodies to r*Pb*MTRAP in reducing fertilisation was first assessed *in vitro*. Purified antibodies obtained from the mice immunised with r*Pb*MTRAP/IFA or from control mice (IFA alone) were added to conversion assays with *Ookluc* in concentrations ranging from 3.12 µg/mL to 500 µg/mL. None of the antibody concentrations were able to reduce the formation of ookinetes (Fig. 1E).

We then decided to modify the protocol and evaluate the blocking ability of circulating antibodies, rather than purified antibodies alone, to reduce ookinete formation in conversion assays. Two weeks after the completion of the immunisation regimen mice from both the control Poly I:C group and the r*Pb*MTRAP/Poly I:C immunised group were challenged with 5,000 asexual forms of *Ookluc P. berghei* parasites (Fig. 2A). The parasitaemia and gametocytaemia were monitored by microscopy, and no significant differences were observed between the r*Pb*MTRAP/Poly I:C immunised group and the control group regarding multiplication of sexual and asexual forms (Fig. 2F-G).

**Fig. 3:**
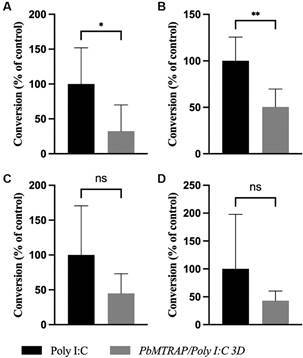
circulating anti-recombinant *Plasmodium berghei* merozoite thrombospondin related anonymous protein (anti-r*Pb*MTRAP) reduce ookinete formation. (A-D) are four independent experiments. Mice were immunised and challenged following the schedule described in Fig. 2A. When the gametocytaemia reached ~0.4%, blood samples from immunised/challenged mice were diluted 1:10 in ookinete medium for conversion assays. The results are expressed as the percentage of ookinete conversion ± standard deviation (SD) relative to the control mean (n = 5 animals per group per experiment). Statistical analyses: unpaired t-test; *p < 0.05, **p < 0.01.

**Fig. 4:**
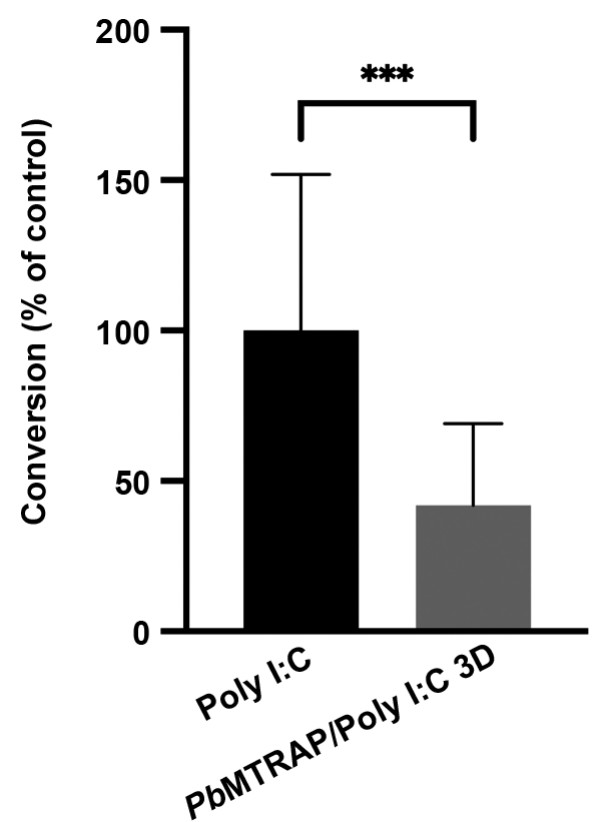
circulating anti-recombinant *Plasmodium berghei* merozoite thrombospondin related anonymous protein (anti-r*Pb*MTRAP) reduce ookinete formation. The four experiments depicted in Fig. 3 were analysed together. The results are expressed as the percentage of ookinete conversion ± standard deviation (SD) relative to the control mean (n = 20 animals per group). Statistical analyses: unpaired t-test; ***p < 0.001.

Once the gametocytaemia reached approximately 0.4%, conversion assays were conducted to assess the blocking activity of the circulating antibodies on ookinete formation in each group of mice. Notably, in two out of four *in vivo* experiments there was a significant reduction of 49% and 83% in ookinete formation in conversion assays using blood from mice immunised with r*Pb*MTRAP/Poly I:C compared to the control group (Fig. 3), and a reduction trend was observed in the other two experiments. When analysed all together, the results of the four independent experiments showed a significant reduction in ookinete formation in conversion assays with blood from mice immunised with r*Pb*MTRAP/Poly I:C (Fig. 4). This significant decrease in ookinete formation strongly suggests the inhibitory effect of *Pb*MTRAP-specific antibodies in a physiological setting.

These findings indicate a potential of the generated antibodies to effectively reduce ookinete formation, further supporting the hypothesis that MTRAP could serve as a target for the development of transmission-blocking interventions.

## DISCUSSION

Implementing TBVs as part of comprehensive malaria control efforts holds great promise in breaking the chain of transmission and ultimately reducing the burden of malaria. The success of the TBVs depends on the capacity to induce neutralising antibodies that remain active in the mosquito midgut against sexual stages of *Plasmodium* and can interfere in the parasite survival, blocking the infection in the vector.^4^ ^,^ ^10^

In this study, we expressed the recombinant *Pb*MTRAP ectodomain in *E. coli* as a heterologous protein, and used it to immunise mice and raise specific antibodies to *Pb*MTRAP. The antibodies were used in conversion assays *in vitro* and *ex vivo* to assess their ability to reduce *P. berghei* fertilisation. The reduction in ookinete formation in conversion assays performed using blood from mice immunised and challenged indicates that MTRAP is a potential target to compose TBV formulations.

An essential requirement for an antigen to be effective as a TBV is its ability to induce robust antibody production even at low vaccine doses, enabling the antibodies to effectively inhibit parasite development in the mosquito midgut.^11^ As recombinant vaccine units generally require addition of an adjuvant to enhance and prolong an adequate immunity, our first studies to test immunogenicity *in vivo* were performed using Freund’s adjuvant, which is known as a powerful stimulator of the immune response *in vivo*. We observed a significant increase in anti-r*Pb*MTRAP antibody titres in the immune serum compared to the control group, demonstrating evident immunogenicity and specificity towards the recombinant antigen. However, the purified anti-r*Pb*MTRAP antibodies did not have activity against the parasite fertilisation *in vitro* at the different concentrations tested. In contrast, antibodies raised in mice immunised with r*Pb*MTRAP formulated with Poly (I:C) were able to reduce ookinete formation in *ex vivo* experiments, in which the conversion assay was performed using blood from immunised and challenged mice.

The differences in functional activity of the antibodies from the two experimental designs may be due to many reasons. First, the adjuvants used are different. While evoking a strong immunological response, Freund’s adjuvant is not suitable for use in humans. Poly (I:C) is a synthetic double-stranded RNA (dsRNA) capable of activating various components of the host defence system in a manner reminiscent of viral infections. Thus, this adjuvant induces a potent interferon response and promotes the expression of various cytokines and chemokines. Such an immune response has demonstrated promising results in conferring protection against malaria in murine models;^12^ second, it is possible that the antibody response induced after immunisations may have been boosted by the challenge, since MTRAP is expressed in circulating sexual and asexual stages of the parasite.^7^ ^,^ ^8^ Such boost may have had an impact both in magnitude and quality of the antibody response, increasing the ability to reduce ookinete formation. Moreover, the challenge determined a prolonged exposure of parasites to circulating antibodies for at least five days. This extended interaction could have facilitated access to the antigen, even during intracellular stages of the parasites, potentially contributing to the observed significant reductions in ookinete formation and conversion;^3^ ^,^ ^13^ third, anti-*Pb*MTRAP activity against ookinete formation may be dependent on complement activity. This is known for at least one transmission-blocking target, P230.^14^ Future experiments using other mouse strains like C57BL/6, that tend to be more inflammatory than BALB/c, may also help explain the role of the blood microenvironment and its components, like cytokines, in the activity against the sexual stages.

It is worth noting that the reduction in ookinete formation in the *ex vivo* experiments occurred in conversion assays in which blood was diluted. The reduction/blockage may be more robust in a natural infection, in which the blood from the host is not highly diluted in the mosquito midgut. Unfortunately, we do not have in Brazil colonies of *Anopheles* species permissive to *P. berghei* infection, so this could not be tested.

Over the past few decades, the battle against malaria has achieved remarkable progress, resulting in significant reduction in both the number of cases and mortality rates. However, the emergence of insecticide-resistant mosquitoes and multidrug-resistant parasites are serious obstacles in the fight against malaria. Developing TBVs will add to the future arsenal to fight malaria. This study provides evidence that MTRAP may be pursued as a target for TBV development.
